# 1385. Granulomatous Mastitis: An Elusive Disease

**DOI:** 10.1093/ofid/ofad500.1222

**Published:** 2023-11-27

**Authors:** Maria Daniela Jarrin Jara, Michael Kladias, Christina Coyle

**Affiliations:** NYC Health + Hospitals/Jacobi, Brooklyn, New York; NYC Health and Hospitals/Jacobi, New York, New York; Albert Einstein College of Medicine, New York, NY

## Abstract

**Background:**

**Background**

Granulomatous mastitis (GM) is a chronic inflammatory disease of the breast characterized by painful and recurrent breast lesions and masses. It affects women of childbearing age with a history of breastfeeding. It is a rare diagnosis with an estimated incidence of 2.4 per 100,000 women in the United States, with a prevalence 12 times higher in Hispanic women.

**Methods:**

**Methods**

We conducted a retrospective review of women with GM seen at NYC Health + Hospitals/Jacobi, a safety net hospital in the Bronx, New York City from January 2017 to March 2023. Patients were included if they had pathologic confirmation of the disease. Demographic and clinical data was extracted from the electronic medical records.

**Results:**

**Results**

A total of 29 patients were included, 86.2% were Hispanic; median age was 40 years old. None of the patients was postpartum and only one patient was breastfeeding at the time of diagnosis. Pain and unilateral palpable mass were the most commonly reported symptoms. Of those who had cultures, only 42.1% reported growth and the most prevalent organisms were *Corynebacterium* species. 89.7% received antibiotic treatment as a first line therapy; only 37.9% of them reported improvement within a month of presentation. Trimethoprim-sulfamethoxazole, clindamycin and doxycycline were the most prescribed treatments. 27.6% of all patients received systemic steroids following failure to improve with antibiotic treatment and 17.2% received intralesional steroids. 65.5% of those reported resolution of symptoms within three months. Two patients received a trial of adalimumab. Seven patients (24%) presented with recurrence after a year.

**Conclusion:**

**Conclusions**

GM occurs predominantly in Hispanic females and seems to be associated with *Corynebacterium* species when an organism is isolated. Recurrences occur in ¼ of patients despite therapy. The pathophysiology of this condition and optimal treatment of this disease is not well defined, but antibiotics alone do not seem to be effective. Immune modulation seems to be an important adjunctive treatment, but which agent and how long is not clear. Further studies are needed to elucidate these answers.

**Disclosures:**

**All Authors**: No reported disclosures

